# Editorial: Interprofessional approaches for the management of chronic diseases

**DOI:** 10.3389/fmed.2024.1490575

**Published:** 2024-09-20

**Authors:** Alberto Marcos Heredia-Rizo, María Jesús Casuso-Holgado, Javier Martínez-Calderón

**Affiliations:** ^1^Instituto de Biomedicina (IBiS) de Sevilla, Departamento de Fisioterapia, Universidad de Sevilla, Sevilla, Spain; ^2^CTS 1110: Understanding Movement and Self in Health From Science (UMSS) Research Group, Andalusia, Spain

**Keywords:** chronic diseases, multidisciplinary, self-efficacy, self-management, prevention

## Background

Chronic diseases, as long-lasting conditions that require ongoing medical attention, can often be controlled, but not cured, and tend to limit daily life activities. These are the leading causes of death and disability, accounting for nearly 75% of all deaths globally (1), and represent the main drivers of increased healthcare costs and decreased productivity. Clinical management of chronic conditions such as cardiorespiratory diseases, stroke, mental health issues, diabetes, or cancer, among others, require an interdisciplinary, collaborative, and person-centered workforce (2). Common modifiable risk factors, including dietary habits, lack of physical activity, and tobacco and alcohol use, are responsible for most of these diseases. Urgent action is needed to support innovative and effective preventive strategies to reduce the associated socioeconomic burden. Toward this, aspects related to promote self-management and self-efficacy can help patients to successfully cope with their conditions and improve their general wellbeing and quality of life.

### Research

This editorial summarizes the contributing articles to the Research Topic “*Interprofessional Approaches for the Management of Chronic Diseases*”. We received a total of 23 manuscripts and one manuscript summary and were able to approve thirteen of them for publication. Out of these papers, eight were original research, two were study protocols, one was an umbrella review, another was a retrospective analysis, and the last paper was a co-creation workshop.

Overall, the included studies highlighted the multifaceted aspects of chronic diseases in diverse populations and clinical settings, reflecting the importance of prevention and individual self-management and the complex relationship between various biopsychosocial factors in the development and persistence of chronic diseases.

Three studies including participants with chronic musculoskeletal conditions assessed the impact of self-efficacy as an outcome measure. Perez-Dominguez et al. translated and culturally adapted the Spanish Version of the Pain Self-Efficacy Questionnaire. This study analyzed 107 patients with rotator cuff injuries and found the questionnaire had good psychometric properties, with high validity, test-retest reliability, and internal consistency. In 68 outpatients with rheumatoid arthritis, a longitudinal study developed by Hayashi et al. observed if discordances between patients and physicians in global assessment may influence pain-related outcomes at 9 years of follow-up. They found that patients who showed a worse perception in their global assessment, in comparison to physicians assessment at baseline, reported higher pain intensity and pain catastrophizing, and lower quality of life and pain self-efficacy at the follow-up. Further, a clinical trial conducted by Fu et al. evaluated the effects of a multidisciplinary treatment plan in patients who received surgery for cervical spondylosis. This study highlighted the benefits of this type of interprofessional treatment in improving important aspects such as neck disability, self-efficacy, quality of life and patient satisfaction, compared with routine care.

Two of the papers were developed as study protocols. Huon et al. proposed a secondary study in primary care by addressing the combined effect of an interprofessional intervention delivered by general practitioners and community pharmacists to deprescribe benzodiazepines and related drugs in older adults. For this purpose, the pharmacists will be trained in motivational interviewing and the impact of the intervention will be measured in terms of acceptability, appropriateness, cost, and fidelity. The second was a protocol of a randomized controlled trial conducted by Martínez-Miranda et al. that aims to evaluate the efficacy of a physiotherapy-focused individualized program using pain neuroscience education and graded exposure to movement in breast cancer survivors. The ultimate goal of this approach was to reinforce individual self-management and empower women throughout cancer care.

A comprehensive overview of current evidence on integrative medicine treatments, e.g., diet, mind-body therapies, and herbal medicine, for chronic, inflammatory, and autoimmune diseases such as rheumatoid arthritis, spondylarthritis, and gout, was provided by Lin et al.. After including 52 reviews, this umbrella review concluded that exercise was effective and, therefore, should be prescribed for this population. Chang et al. delved into nurse-led case management in addition to usual care in 96 adults with rheumatoid arthritis. Results from the study demonstrated that the multimodal approach was helpful to reduce pain and fatigue, offering a new perspective to provide tailored care for these individuals. From a preventive viewpoint, Zhang et al. explored the predictive role of visceral adipose tissue in people with osteoarthritis using a two-sample Mendelian randomization method. Their findings suggested that decreasing the accumulation of visceral adipose tissue could reduce the incidence of osteoarthritis, whether at the hip, knee, or spine. In a sample of 112 individuals with ankylosing spondylitis, Cortes-Rodríguez et al. investigated the impact of foot health on the quality of life. They observed that adults with ankylosing spondylitis present lower quality of life concerning foot health and overall wellbeing compared to healthy population, which highlights the importance of a podiatrist involvement for regular foot checks.

In a retrospective analysis of 411 patients with fibromyalgia, Rivera et al. investigated the possible association of different comorbidities with functionality, pain, and depression in this cohort. Obesity was the most robust significant predictor since obese patients were twice more likely to have poorer functional status. The authors suggest that it is important to address obesity in people with fibromyalgia in order to manage this condition. Kassaw et al. conducted a hospital-based cross-sectional study to investigate whether medication regimen complexity was associated with medication adherence in patients with multimorbidity. They found that nearly half of the 416 participants had low medication adherence, and that medication complexity was negatively associated with medication adherence, thus healthcare providers should develop effective strategies to improve this lack of adherence to pharmacological treatments in people with multiple chronic diseases. Exercise is a key strategy for non-communicable chronic diseases, but the problem of low levels of physical activity in adults with intellectual disability is well-recognized. Kwan et al. conducted a multicentre controlled trial to examine the effectiveness of a video-based exercise programme in promoting physical activity in this population. They included 160 participants who underwent an 8-week exercise intervention and observed positive changes in the physical condition. Finally, chronic pain, a health condition that requires a biopsychosocial approach and a person-centered interprofessional care, was investigated by Berryman et al.. These authors have developed a co-creation workshop to generate ideas about how to provide evidence-based and efficient care in a pediatric chronic pain service. Thirty-four multidisciplinary skateholders participated in this collaborative activity and provided a launch pad for innovative clinical and research ideas. This study highlights the importance of creating interprofessional networks to improve the management of chronic conditions.

## Summary

To summarize, chronic diseases have a multifactorial etiology, which makes them difficult to manage in the clinical setting. Strengthening the collaboration among healthcare professionals, while always respecting individual differences among professionals and patients, is likely the best approach to provide an evidence-based response to the preventive and therapeutic treatment of chronic diseases.
